# Incorporating CNV analysis improves the yield of exome sequencing for rare monogenic disorders—an important consideration for resource-constrained settings

**DOI:** 10.3389/fgene.2023.1277784

**Published:** 2023-12-14

**Authors:** Nadja Louw, Nadia Carstens, Zané Lombard, for DDD-Africa as members of the H3Africa Consortium

**Affiliations:** ^1^ Division of Human Genetics, National Health Laboratory Service and School of Pathology, Faculty of Health Sciences, University of the Witwatersrand, Johannesburg, South Africa; ^2^ Genomics Platform, South African Medical Research Council, Cape Town, South Africa

**Keywords:** copy number variation, exome sequencing, low-middle-income countries, variant calling, rare disease, monogenic disorders

## Abstract

Exome sequencing (ES) is a recommended first-tier diagnostic test for many rare monogenic diseases. It allows for the detection of both single-nucleotide variants (SNVs) and copy number variants (CNVs) in coding exonic regions of the genome in a single test, and this dual analysis is a valuable approach, especially in limited resource settings. Single-nucleotide variants are well studied; however, the incorporation of copy number variant analysis tools into variant calling pipelines has not been implemented yet as a routine diagnostic test, and chromosomal microarray is still more widely used to detect copy number variants. Research shows that combined single and copy number variant analysis can lead to a diagnostic yield of up to 58%, increasing the yield with as much as 18% from the single-nucleotide variant only pipeline. Importantly, this is achieved with the consideration of computational costs only, without incurring any additional sequencing costs. This mini review provides an overview of copy number variant analysis from exome data and what the current recommendations are for this type of analysis. We also present an overview on rare monogenic disease research standard practices in resource-limited settings. We present evidence that integrating copy number variant detection tools into a standard exome sequencing analysis pipeline improves diagnostic yield and should be considered a significantly beneficial addition, with relatively low-cost implications. Routine implementation in underrepresented populations and limited resource settings will promote generation and sharing of CNV datasets and provide momentum to build core centers for this niche within genomic medicine.

## 1 Introduction

Exome sequencing (ES) is a widely used molecular approach and is recommended as a first-tier test for diagnostic purposes for rare monogenic disorders (Stark et al., 2016; Hu et al., 2018; Srivastava et al., 2019). ES identifies variants within coding exonic regions and is predominantly centered around single-nucleotide variant (SNV) discovery. Recent computational advances have made it possible to incorporate copy number variant (CNV) analysis from ES data, making it more practical and cost-effective, especially for disorders where both SNVs and CNVs are involved in disease etiology.

CNVs attribute to the pathogenesis of up to 15% of rare monogenic cases (Truty et al., 2019; Testard et al., 2022) and tend to have a more severe consequence on phenotype compared to SNVs due to their large size and effect on entire coding regions (Park et al., 2019). Progress has been made regarding joint SNV and CNV investigations in low-middle-income countries (LMICs); however, the gold standard for CNV detection remains chromosomal microarray (CMA) despite its inability to detect SNVs or smaller insertions and deletions. Thus, CMA does not facilitate the efficient use of resources when applied exclusively within already resource-limited settings (Park et al., 2019).

ES has proven to be a cost-effective first-tier test in developed countries, predicting a cost saving between $1,484 and $3,242 per diagnosis (Schwarze et al., 2018). Implementing a diagnostic exome is still thought to be higher in LMICs due to the lack of established infrastructure, high cost of reagents, and the need for personnel training (Wiener et al., 2023); however, studies show that traditional genetic testing and pre-ES investigations can cost up to six times more than local ES costs (Cordoba et al., 2018; Masri and Hamamy, 2021). ES as a first-line investigation would thus be beneficial for many patients and a worthwhile investment in a limited resource setting (Wiener et al., 2023). Despite the advances in ES, it is still not routinely used, especially in countries where genetic testing is limited. The overall diagnostic rate of ES is estimated at ∼25% (Yang et al., 2013; Lee et al., 2014; Fung et al., 2020); however, yields as high as 36% (Srivastava et al., 2019) and 41% (Dong et al., 2020; Wright et al., 2023) have been reported in patients with rare monogenic developmental disorders. In studies involving consanguineous patients, a yield of up to 86% has been reported (Hiz Kurul et al., 2022).

In this mini review, we present evidence to show that integrating CNV detection tools into a standard ES analysis pipeline should be considered since it is cost-effective, improves diagnostic yield, and shortens the diagnostic odyssey for patients.

## 2 Bioinformatic considerations and CNV calling tools

**FIGURE 1:**
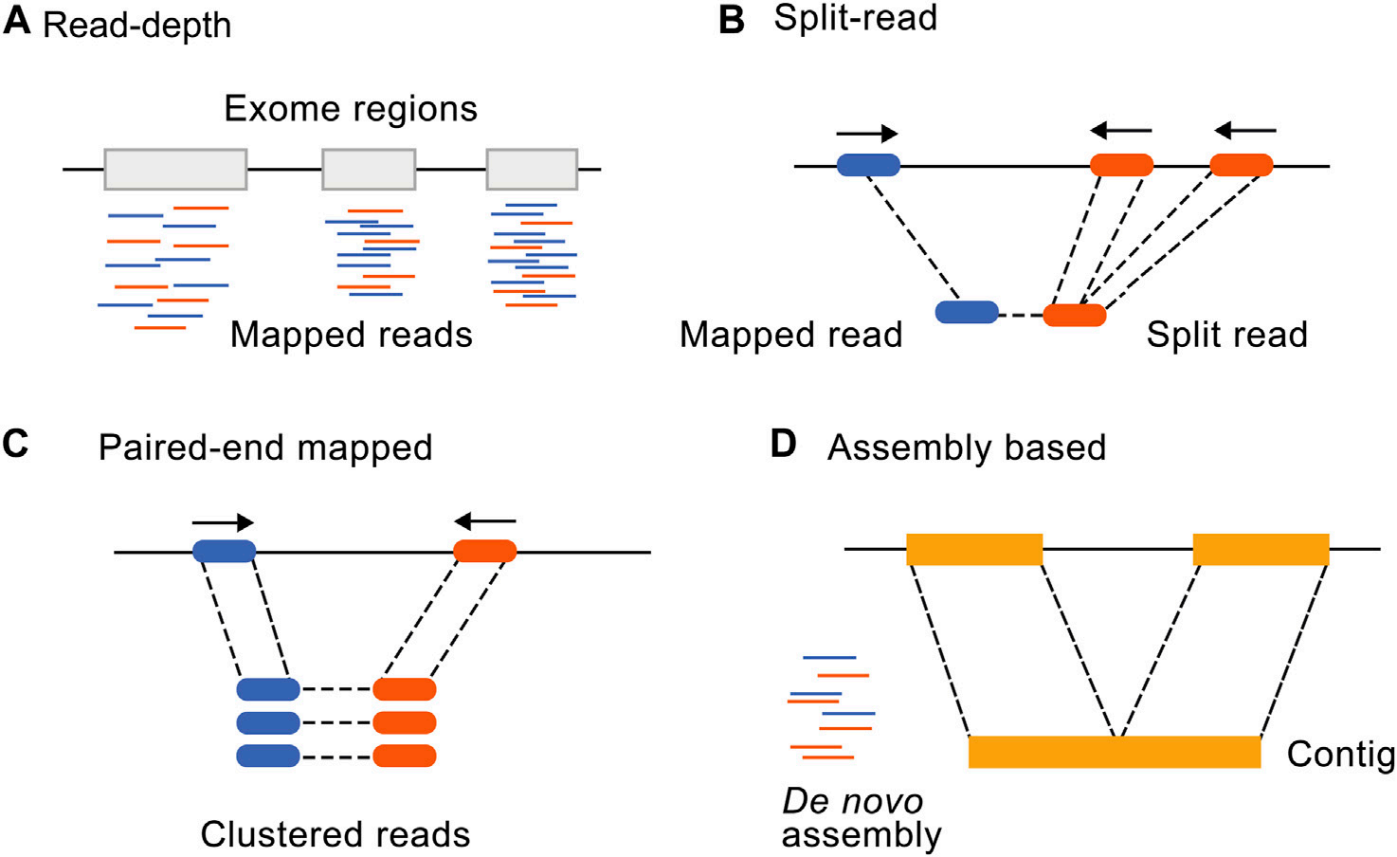
Illustration of the four main CNV calling methods from NGS data. Adapted from Zhao et al. (2013), licensed under CC BY 2.0. **(A)** Read depth-based, **(B)** Split read-based, **(C)** Paired-end mapping, and **(D)** assembly-based approaches.

Many bioinformatic tools have been developed for the identification of CNVs from genome and ES data. While no additional sequencing costs are involved in the exome CNV analysis, computational costs relating to additional data analysis should be considered. Comparing computational costs of exome CNV tools, the average expected central processing unit usage was 5.68 GHz and an average of 267,55 Mb of space was used for a 11.2 Mb series of datasets with ×100 coverage (Zhao et al., 2020). Implementing CANOES on 285 samples took ∼6 min per sample using a 2.3 GHz central processing unit core (Backenroth et al., 2014) and for CLAMMS, (Packer et al., 2016) an estimate of ∼50 MB random-access memory is required per process. The four main approaches to detect CNVs (Figure 1) are read depth-based, paired-end mapping, split read-based, and assembly-based approaches (Zhao et al., 2013). A combination or ensemble approach is also commonly used as none of the methods alone detect all CNVs with high specificity and sensitivity. Here, we will focus on the most recent and most widely used CNV tools (Gabrielaite et al., 2021) divided into categories according to these different detection approaches (Table 1).

**TABLE 1:** Summary of bioinformatic tools for CNV detection using next-generation sequencing data.

Tool	Method	Advantages/Attributes	Reference
CANOES	Read depth	Rare CNVs from ES data	Backenroth et al. (2014)
CLAMMS	Read depth	Large population ES studies and easy integration into automated variant-calling pipeline	Packer et al. (2016)
CoNIFER	Read depth	More insertions identified and low memory required. ES-specific tool	Krumm et al. (2012)
ExomeDepth	Read depth	Good control of technical variability between samples and effective across a wider range of exome datasets. Small and heterozygous deletions	Plagnol et al. (2012)
XHMM	Read depth	Explores novel classes of CNVs from ES data	Fromer et al. (2012)
Cn.MOPS	Read depth	Larger CNVs identified from ES and WGS data. Fast average running time	Klambauer et al. (2012)
GATK-gCNV	Read depth	Detects rare CNVs and determines copy number biases and CNVs from WGS and ES data	Babadi et al. (2022)
HMZDelFinder/HMZDelFinder _opt	Read depth	Rare, intragenic homozygous and hemizygous deletions from ES data	Gambin et al. (2017)
EXCAVATOR2	Read depth	Detects CNVs from ES data with genome-wide resolution	D’aurizio et al., 2016
CODEX	Read depth	No matched normal controls required. Designed for ES data.	Jiang et al. (2012)
CN-Learn	Read depth	Small sample size needed, good precision, and high-confidence CNVs from ES data	Pounraja et al. (2019)
CNVkit	Read depth	<100 kb CNVs from WGS and ES data and more effective for deletions	Talevich et al. (2016b)
PINDEL	Split read	Large deletions and medium-sized insertions from WGS	Ye et al. (2018)
PRISM	Split read	SVs and precise breakpoints from WGS	Jiang et al. (2012)
SVseq2	Split read	Indel from low coverage WGS data and exact breakpoints	Zhang et al. (2012)
Gustaf	Split read	Identifies size and location of dispersed duplications and translocations from WGS data; 30–100 bp and >500 bp	Trappe et al. (2014)
INDELible	Split read	Smaller SVs (20–500 bps) from ES data missed by conventional methods	Gardner et al. (2021)
BreakDancer	Paired-end	Various SVs including indels, inversions, and translocations from WGS	Chen et al. (2016)
HYDRA	Paired-end	Diverse classes of SVs, including those involving repetitive elements such as transposons and segmental duplications from WGS	Quinlan et al. (2010)
PEMer	Paired-end	∼3 kilobases or larger SVs from WGS	Korbel et al. (2009)
Ulysses	Paired-end	High specificity over a complete spectrum of variants	Gillet-Markowska et al. (2015)
Magnolya	Assembly based	No mapping of reads to reference genome and *de novo* CNV detection	Nijkamp et al. (2012)
DELLY	Ensemble approach	Full spectrum of genomic rearrangements from WGS, including complex events	Rausch et al. (2012)
LUMPY	Ensemble approach	Increased sensitivity of SV detection from WGS	Layer et al. (2014)
Manta	Ensemble approach	Less computational time/space, intense large-scale SVs, medium-sized indels, and large insertions from WGS and ES data	Chen et al. (2016)
GenomeSTRiP	Ensemble approach	Whole-genome discovery and genotyping of deletions	Handsaker et al. (2011)
CNVer	Ensemble approach	Better mitigates the sequencing biases causing uneven local coverage and accurately predicts CNVs	Medvedev et al. (2010)

*Whole-genome sequencing (WGS).

*Structural variation (SV).

### 2.1 Read depth-based approach

This approach relies on the depth of coverage to estimate the copy number that the genomic region is correlated with. A higher depth of coverage at a specific region indicates a gain, whereas a lower depth of coverage indicates a loss of copy number. This approach performs well in complex genomic regions (Yoon et al., 2009). CLAMMS (Packer et al., 2016), CoNIFER (RRID:SCR_013213) (Krumm et al., 2012), ExomeDepth (RRID:SCR_002663) (Plagnol et al., 2012), XHMM (Fromer et al., 2012), cn.MOPS (RRID:SCR_013036) (Klambauer et al., 2012), and GATK-gCNV (Babadi et al., 2022) are amongst the most recent and often used read depth-based tools. As only the exonic regions are sequenced, some considerations need to be addressed for these tools to function optimally. ES is considered more appropriate for this approach since it has higher coverage than whole genome sequencing. The majority of tools developed to date for the identification of CNVs from ES data are thus based on this approach. When using read depth-based CNV detection, one should take into consideration that most tools require the use of a reference panel of samples. An assumption of the read depth approach is that reads are distributed uniformly across the genome; however, this is not the case for exome sequencing. Reference samples are thus used to control these biases created by regions of variable depth across exons by establishing a baseline for CNV calling, which ensures the accurate detection of CNVs. These samples should ideally be matched in terms of preparation and sequencing platform and even sequencing batch if possible to limit technical biases which might hinder CNV detection. A number of tools require matched case–control samples as inputs; however, many tools use multiple test samples as a cohort to serve as reference samples for the analysis. The number of samples to be used ranges from below ten to hundreds of samples; for instance, cn.MOPS requires a minimum of six samples, whereas XHMM has a minimum of 50 samples. Several read depth-based tools have been developed and implemented on ES data (Krumm et al., 2012; Tan et al., 2014; Pfundt et al., 2017; Zhao et al., 2020; Gordeeva et al., 2021).

### 2.2 Split read-based approach

This approach detects unmatched read pairs; thus, one read aligns to the reference genome, while the other read fails to map or aligns only partially to the genome. This potentially identifies the breakpoints for CNVs. A few recent tools developed based on this approach are PINDEL (RRID:SCR_000560) (Ye et al., 2018), PRISM (RRID:SCR_005375) (Jiang et al., 2012), SVseq2 (Zhang et al., 2012), and Gustaf (Trappe et al., 2014). One tool developed specifically for ES data is INDELible (Gardner et al., 2021) which was designed to target smaller structural variations (21–500 bp) mostly missed by other CNV calling tools.

### 2.3 Paired-end mapping approach

This approach was the first approach to put forth the possibility of using next-generation sequencing (NGS) data to detect CNVs (Tuzun et al., 2005; Korbel et al., 2009). It relies on the insert size from the library preparation process and identifies any decreased insert size or swapped read directions between read pairs to identify a CNV or mobile element, insertions, inversions, and tandem duplications. In regions of low complexity containing segmental duplications, this approach seems to be limited. A number of tools have been developed, such as BreakDancer (RRID:SCR_001799) (Fan et al., 2014), HYDRA (RRID:SCR_005260) (Quinlan et al., 2010), PEMer (RRID:SCR_005263) (Korbel et al., 2009), and Ulysses (Gillet-Markowska et al., 2015) being the most widely used (Zhao et al., 2013; Gabrielaite et al., 2021).

### 2.4 Assembly-based approach

The assembly-based approach assembles reads *de novo* and does not align to a reference genome. Overlapping reads are assembled, and these contigs are then compared to the reference genome, identifying regions with contradictory copy numbers. A minimum read coverage is required for tools based on this approach to be used successfully. The most commonly used assembly-based tool is Magnolya (RRID:SCR_000164) (Nijkamp et al., 2012).

### 2.5 The ensemble approach

None of the abovementioned methods alone detects the full spectrum of CNVs with high sensitivity and specificity, and thus it is recommended to use an ensemble approach. In this regard, several tools have been developed to integrate multiple approaches and increase performance. These include DELLY (RRID:SCR_004603) (Rausch et al., 2012), LUMPY (RRID:SCR_003253) (Layer et al., 2014), Manta (Chen et al., 2016), CNVer (RRID:SCR_010820) (Medvedev et al., 2010), and GenomeSTRiP (Handsaker et al., 2011). Although this approach is recommended, there is still no gold standard for CNV detection, especially from ES data. A review of recent publications making use of these bioinformatic tools will thus provide a clearer indication of what approach to consider when calling CNVs from ES data.

## 3 Best approaches for CNV calling from ES data

As there are many tools available for CNV detection from ES data, recommendations have been made focusing on the use of these tools for optimal results. In a recent comparative analysis of ES-focused CNV tools (Zhao et al., 2020), the recommendations for obtaining the best results were related to the specific dataset. In terms of accuracy, it was recommended to use CNVkit (Talevich et al., 2016) if CNV size is small (<100 kb), whereas cn.MOPS seems to be optimal if CNV size is larger. If the dataset presents with more insertions, using CoNIFER is recommended, and CNVkit is seemingly the best for identifying deletions. If there is no prior knowledge of the dataset, then using cn.MOPS and CoNIFER together is recommended (Guo et al., 2013; Zhao et al., 2020).

Different tools have been designed to obtain optimal sensitivity and specificity focused on rare or common CNVs as well as population size. Previous limitations, for instance, only being able to identify CNVs spanning at least two or more exons, GC content, or mappability biases as well as sequencing noise have been addressed, and many tools have been developed to try and overcome these difficulties (Pounraja et al., 2019; Bigio et al., 2020; Filer et al., 2021; Babadi et al., 2022). CLAMMS was developed to be more suitable for large population studies (Packer et al., 2016) and integrated more easily into an automated variant calling pipeline. In order to more accurately identify rare and intragenic homozygous and hemizygous deletions, HMZDelFinder (Gambin et al., 2017) was developed, and the newer HMZDelFinder_opt (Bigio et al., 2020) outperforms the older version in terms of accuracy and specifically identifies partial exon deletions. ExomeDepth has also been widely used (Marchuk et al., 2018; Rajagopalan et al., 2020; Zhai et al., 2021) and designed to control technical variability between samples. CANOES (Backenroth et al., 2014) is complementary to methods like XHMM and CoNIFER, and accuracy can be improved when using CANOES in combination with one of these methods. CN-Learn identifies true CNVs with higher precision and recall rates without compromising performance even with as little as 30 samples (Pounraja et al., 2019). This tool uses CNVs predicted by four different CNV callers (CANOES, CODEX, XHMM, and CLAMMS) which were found to enhance performance instead of using the tools as standalone methods. Another study also merged results from CANOES and HMZDelFinder after each tool was applied separately (Dong et al., 2020). It was also suggested to combine GATK-gCNV, LUMPY, DELLY, and cn.MOPS which have the best recall and capture different CNVs (Gabrielaite et al., 2021). While LUMPY and DELLY have been developed for whole-genome sequencing data, GATK-gCNV and cn.MOPS should be used with ES data. In a recent study, CNVkit, XHMM, EXCAVATOR2, and ExomeDepth were used for ES-based CNV calling in order to maximize the sensitivity and make ES a more powerful tool to diagnose neurodevelopmental disorders (Zhai et al., 2021).

It is clearly demonstrated that the ensemble approach yields optimal results while increasing the sensitivity and specificity of CNV detection (Välipakka et al., 2020). Individual implementation strategies could still be helpful and lead to an increased diagnostic yield but is largely influenced by the available computing infrastructure in specific environments as well as adequate representation of the different calling strategies. CNV calling from ES data should be particularly attractive in resource-constrained settings with reduced capital expenditure and required infrastructure.

## 4 Value of CNV calling from ES in resource-constrained countries

In a recent study (Dong et al., 2020), an overall yield of 41.4% was reported by the simultaneous analysis of SNV and CNV, of which 12.0% can be attributed to CNVs. Another study based in China found that SNV and exome-based CNV calling yielded an overall diagnostic rate of 58.8%, of which diagnostic CNVs accounted for 17.6% (Xiang et al., 2021). A comprehensive method was used for CNV identification which included combining XHMM and principal component analysis with CNVKit. Similarly, it was found that incorporating exome-based CNV detection into conventional SNV analysis for a single trio-ES test significantly improved the diagnostic rate (Zhai et al., 2021). When combining SNV and CNV analyses, an overall diagnostic yield of 54% was obtained, which included 18.9% from CNV analysis alone. CNVs in this study were detected using CNVkit, XHMM, EXCAVATOR2, and ExomeDepth, which were all retained and annotated thereafter.

**FIGURE 2:**
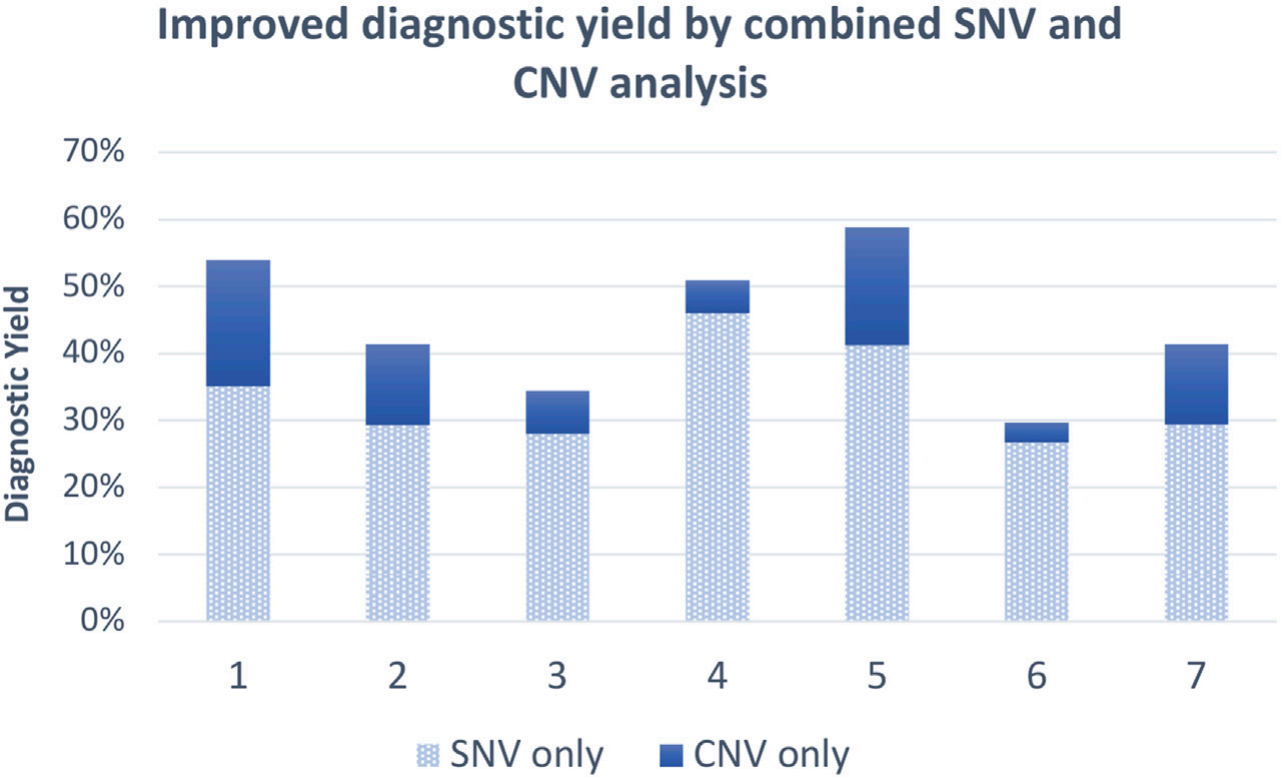
Average diagnostic yield of exome SNVs and combined SNVs/CNVs from 1. Zhai et al. (2021), 2. Truty et al. (2019), 3. Pranav Chand et al. (2023), 4. Moosa et al. (2022), 5. Xiang et al. (2021), 6. Sheth et al. (2023), and 7. Dong et al. (2020).

In an effort to identify the cause of congenital heart disease in 96 child participants from Nigeria, a combined approach was applied by making use of ES from patient and parents (where available) and performing XHMM CNV analysis on the data (Ekure et al., 2021). Assessing the genomic etiology of autism spectrum disorder in India, a diagnostic yield of 29.7% of individuals in total was obtained for exome sequencing, of which CNVs contributed 3%, and interestingly, CMA analysis carried out on the same cohort yielded a diagnostic rate of 2.9% (Sheth et al., 2023). Thus, combined CNV and SNV analysis from ES data significantly increased the diagnostic yield versus only using CMA (29.7% vs. 2.9%). The combined SNV and CNV analysis from the discussed literature has been shown to increase the diagnostic yield by as much as 18% (Figure 2), which is an additional diagnosis for ∼2 out of every 100 individuals. The average increased yield attributed to CNVs from the discussed research is 10.7% without additional testing costs involved. Therefore, implementing ES as a first tier for diagnosis, especially when incorporating CNV analysis, should be considered because it is efficient and cost-effective and shortens the diagnostic odyssey for patients who would not have otherwise necessarily received a molecular diagnosis.

As is the case for most resource-limited settings, the cost of sequencing a trio and availability of both parents are always the important limiting factors. A study carried out in India (Pranav Chand et al., 2023) on children with neurodevelopmental delay found that a proband-only ES approach obtained a diagnostic yield of 31.5% of these children. Addition of parental samples increased this yield by only 3%, and CNVs contributed to 6.5% of the diagnoses. Another study conducted in China had an overall diagnostic rate of 28.8% after analyzing 1,323 pediatric patients, which proved to be a relatively efficient and cost-effective approach in a developing country (Hu et al., 2018). A South African study (Moosa et al., 2022) found that proband-only ES is a very valuable tool for diagnosis, especially if CNV analysis is included. A diagnostic yield of 51% was obtained with 46% of patients presenting with SNVs and 5% with CNVs. Even though trio-ES has been shown to have the best outcome for a positive diagnosis (Wright et al., 2023), proband-only exome analysis has proven to be a feasible option for diagnosis in settings with limited resources or difficulty in obtaining parental samples.

Another advantage of identifying CNVs in underrepresented populations is the expansion of variant representation in predominantly European-focused public data repositories. Recent progress has been made to contribute CNVs from African population groups to variant databases (Nyangiri et al., 2020; Romdhane et al., 2021; Yilmaz et al., 2021) as the lack of diversity of high-quality genomic data, specifically from Africa, hampers the implementation of appropriate genetic services and brings forth healthcare inequalities (Baine-Savanhu et al., 2023). The lack of representation in population frequency databases has also made clinical interpretation and classification of CNVs more challenging in LMICs. Standardized CNV reporting is possible by using specific ACMG and Clinical Genome (ClinGen) Resource guidelines for CNV classification (Riggs et al., 2020), but careful evaluation of CNVs is encouraged to ensure that only likely disease-causing CNVs matching the patient phenotype are reported. Resolving VUSs remains challenging if population representation is inadequate. Furthermore, additional investigations including validation and functional experiments are often not available in LMIC laboratories. Distinguishing benign CNVs from pathogenic CNVs can be challenging, and thus a number of tools have been developed to enable a more convenient manner of CNV annotation and interpretation. These tools provide support for annotation and/or classification of CNVs, and many tools are web-based, easy to use, and freely available. A recent review have summed up these tools comprehensively to make it easier for clinicians, laboratory scientists, and genetic counselors to make a decision as to which tool would work best in their setting (Pös et al., 2021).

## 5 Discussion

Overall, simultaneous analysis of CNVs and SNVs through ES shows potential as a first-tier investigation for diagnosing rare monogenic disorders. Novel candidate genes and variants have been identified, representing the first step in genomic studies within understudied populations. The diagnostic yield of the current gold standard for CNV detection (CMA) is 15%–20% (Miller et al., 2010), which is significantly lower than ES. In a recent scoping review, it was shown that ES for diagnosing neurodevelopmental disorders outperforms CMA by 10%–28% (Srivastava et al., 2019), further supporting the combined SNV and CNV analysis approach from ES data. Although it would be ideal to validate exome CNVs with methods such as microarray, it is costly and often not feasible in LMICs. Accurate CNV calling incorporating thorough quality control can help limit false-positive and false-negative results. This is evidenced by eliminating the need for Sanger sequencing validation of SNVs when proper quality control is carried out (Strom et al., 2014). It is also important to note that ES CNV tools have limitations due to their inability to detect specific types of variations, for instance, balanced structural variants (translocations and inversions), mosaicism, and smaller CNVs (<50 bp). Although analyses and technologies are improving to address these shortcomings, it should be considered when implementing these tools. Whole-genome sequencing and array-based techniques can be used to identify these variations; however, this will incur additional costs. Long-read sequencing has the ability to detect these structural variations as well as SNVs, making it ideal to implement as a single assay to replace all the above methods. At present, this technology is too expensive to use as a first-tier test; however, as costs decrease, this might be a possibility for future consideration.

It is evident that a more diverse reference genome representing a larger range of population groups is required to improve CNV calling and classification. Improved diversity in population frequency databases will also provide access to key data needed for the clinical interpretation of CNVs. Although there is still limited data and genetic services in most of Africa, making it difficult to translate research into clinical healthcare services (Kamp et al., 2021), a current and thorough analysis of the cost-benefit for ES would be beneficial toward motivating the adoption of ES as a first-tier test in resource-constrained environments. This review highlights the need for incorporating not only efficient but appropriate exome pipelines in LMICs to further implement genomic medicine and make it more attainable for all. A wider adoption of CNV calling from ES data and use over time will allow for more opportunities to achieve this. Reanalysis of data should be considered for patients without a definite diagnosis as this has proven to increase diagnostic yield (Liu et al., 2019; Schuermans et al., 2022). More CNV publications and ClinVar submissions (Landrum et al., 2014; Landrum et al., 2016; Landrum et al., 2017) from understudied populations will expand the size and scope and improve the resolution of clinically relevant CNVs in the public domain. Public data repositories like ClinVar and DECIPHER (Bragin et al., 2014) have contributed to diversifying data; however, more effective production and sharing of genomic datasets are needed to advance genomic medicine globally. Recent initiatives have been established to facilitate African data-sharing and empower health experts by availing tools, training, and coordination to strengthen laboratory and bioinformatic capacity (Mulder et al., 2016; Mulder et al., 2018; Makoni, 2020; Lumaka et al., 2022). International collaborations and training could be crucial to resolve the true impact of CNVs and build strong core groups with expertise, experience, and technical competence to accurately report on CNVs in a diagnostic context within LMICs. CNV calling from existing ES datasets from non-European individuals may therefore be an important analysis to invest in.
